# Influence of Workload, Personality, and Psychological Flexibility on Occupational Stress Among Medical Staff: A Fuzzy-Set Qualitative Comparative Analysis

**DOI:** 10.3389/fpubh.2022.929683

**Published:** 2022-07-15

**Authors:** Liming Quan, Yang Zhang, Fugui Jiang, Ying Liu, Yajia Lan, Lei Huang

**Affiliations:** ^1^Department of Environmental Health and Occupational Medicine, West China School of Public Health and West China Fourth Hospital, Sichuan University, Chengdu, China; ^2^Cochrane China Center, West China Hospital, Sichuan University, Chengdu, China; ^3^Chinese Evidence-Based Medicine Center, West China Hospital, Sichuan University, Chengdu, China; ^4^Sichuan Provincial Center for Mental Health, Sichuan Academy of Medical Sciences and Sichuan Provincial People's Hospital, Chengdu, China; ^5^West China School of Nursing, West China Hospital, Sichuan University, Chengdu, China; ^6^Department of Occupational Hazard Assessment, West China School of Public Health and West China Fourth Hospital, Sichuan University, Chengdu, China

**Keywords:** medical staff, occupational stress, fuzzy-set qualitative comparative analysis (fs-QCA), workload, personality, psychological flexibility

## Abstract

**Objective:**

During the COVID-19 pandemic, the occupational stress of medical staff has been a major issue. This study aimed to suggest a new strategy to identify high-risk factor sets of occupational stress in medical staff using fuzzy-set qualitative comparative analysis (fs-QCA) and provide ideas for the prevention and intervention of occupational stress.

**Methods:**

A total of 1,928 medical staff members were surveyed and tested using the Acceptance and Action Questionnaire-II (AAQ-II), Occupational Stress Inventory-Revised edition (OSI-R), and Eysenck Personality Questionnaire-Revised Short Scale (EPQ-RSC). The fs-QCA was used to explore the high-risk factors for occupational stress among medical staff.

**Results:**

The psychological strain (PSY) score of the medical staff was 26.8 ± 7.13, and the physical strain (PHS) score was 24.3 ± 6.50. Low psychological flexibility score-introversion-high role overload, introversion-neuroticism-high role overload, and low psychological flexibility score-neuroticism were high-risk factor sets for PSY. Low psychological flexibility score-introversion-high role overload, low psychological flexibility score-introversion-neuroticism, low psychological flexibility score-neuroticism-high role overload, low psychological flexibility score-psychoticism-neuroticism, and psychoticism-neuroticism-high role overload were high-risk factor sets for PHS.

**Conclusion:**

There are different combinations of high-risk factors for occupational stress among the medical staff. For occupational stress intervention and psychological counseling, targeted and individualized health intervention measures should be implemented according to specific characteristic combinations of different individuals.

## Introduction

Stress is a health problem commonly faced by occupational groups, and medical staff are a high-risk group for mental health problems (1–3). A cohort study published in 2018 reported that the prevalence of depression, anxiety, and stress among Australian nurses was reported as 32.4, 41.2, and 41.2%, respectively (4). In addition, a cross-sectional survey conducted in China found that 68.3% of nurses reported high levels of occupational stress (5). A meta-analysis reported that the prevalence of burnout syndrome was highest among nurses, younger persons, and trainees (6). During the COVID-19 pandemic, the situation of the medical staff has been more complex and severe. It is reported that depression and anxiety of workers who had unprotected contact with infectious patients was reported a significant increase in the estimated risk compared to control (7, 8). Francesco et al. (9) found that a significant proportion of workers in emergency care reported more severe burnout symptoms than those engaged in non-healthcare social and administrative duties. In the first wave of COVID-19 pandemic, the prevalence of burnout syndrome in voluntary psychologists was nearly 17%, and neuroticism was positively associated with burnout symptoms (10, 11). In addition, a meta-analysis found that the overall prevalence of occupational stress among medical staff caring for patients with COVID-19 was 45% (12).

Many factors are associated with occupational stress, and work patterns are complex. Stress is not only closely related to working conditions in the occupational environment but is also related to individual characteristics, personality traits, and sociopsychological factors (13–16). Reportedly, individuals with a type A personality (more self-motivated, self-confidence, aggressive, and a sense of achievement) are more likely to suffer from occupational stress (17). Several studies have also found that medical staff and older adults with introverted personalities and negative emotions usually have more severe stress symptoms than control groups (18, 19). In addition, poor psychological resilience, self-efficacy, psychological flexibility, and coping resources are also believed to be related to occupational stress (14, 20). Faced with such a complicated situation, the quick identification of high-risk factors or high-risk factor sets to take targeted intervention measures is the key point for preventing occupational stress.

In previous studies, we focused on the individual effects of target factors, such as social support, self-efficacy, and workload, after adjusting for other variables, such as demographic characteristics (14, 18, 20). However, in most actual situations, several risk factors may exist simultaneously, such as introverted nurses who lack social support but are engaged in high-load work or individuals with low self-efficacy engaged in high-load work. The combination of different traits is complex and variable for different individuals. Identifying the risk of stress among different individuals in occupational activities and what combination of different traits are more likely to cause occupational stress are the issues we want to discuss.

Qualitative comparative analysis (QCA) is a suitable tool to solve this problem. QCA is a data processing method between probability statistical analysis and single-case analysis, and was first launched by Charles Ragin in 1987 (21). It was originally developed in the fields of political science and historical sociology to determine which intervention or combination of interventions might be the most effective (22). QCA brings together qualitative and quantitative data derived from cases to identify the necessary and sufficient conditions for an outcome (23). It has unique advantages in the analysis of complex social problems formed by multiple concurrent causalities (24, 25). QCA subsequently developed a class of models, including clear-set qualitative comparative analysis (cs-QCA), multivalued qualitative comparative analysis (mv-QCA), and fuzzy-set qualitative comparative analysis (fs-QCA) (21, 26). In recent years, fs-QCA has been actively applied in the field of public health. Initially, it was used to evaluate public health interventions, including mental health interventions (27), medication adherence interventions (28) and health promotion interventions (29, 30). At the same time, the fs-QCA also has applications in the discussion of risk factors, such as children's language barriers (31), nurses' ability (32), and adolescent self-esteem and life satisfaction (33). However, no relevant research has been conducted on the interventions and influencing factors of occupational stress.

Occupational stress is a physical and mental health problem caused by multiple factors such as working conditions, personality, and social psychological factors. In this study, we used the fs-QCA method to analyze the effects of role overload, personality, and psychological flexibility on occupational stress among medical staff and explore high-risk factor sets of occupational stress. This study aimed to provide a theoretical basis for effectively coping with occupational stress and formulating intervention measures.

## Materials and Methods

### Study Population

This study used a cross-sectional research design. A self-administered questionnaire and three standardized scales were used to collect data on basic characteristics, personality characteristics, psychological flexibility, and occupational stress from 15 public hospitals. The inclusion criteria were as follows: medical staff in public hospitals above the municipal level; participants including doctors, medical technicians, and nurses; at least 18 years of age; and at least 1 year of service. The exclusion criteria were temporary staff or trainees, logistical administrative staff, retired staff, and rehired staff. The survey was jointly organized by the research group and the hospital personnel department. Specially trained investigators were assigned to different clinical departments to carry out investigations. The task of investigators was to distribute questionnaires and guide the completion of the questionnaires. A total of 1,928 medical staffs were invited as participants using multistage random cluster sampling. During the study, 127 subjects were additionally excluded (withdrew or did not complete the questionnaire and test), and 1,801 subjects were finally included. The ratio of responders over invitations was 93.4%. This study was approved by the Ethics Committee of the West China School of Public Health, Sichuan University (No. Gwll2021070).

### Outcome Measurements

Personality was measured using the Eysenck Personality Questionnaire-Revised Short Scale for Chinese (EPQ-RSC), which includes three dimensions: extraversion, neuroticism, and psychoticism (34). In the extraversion dimension, high scores indicate extroversion (E) and low scores indicate introversion (e). A high score for neuroticism means more emotional reactions such as anxiety and worry, and even irrational behavior (N), while low scores indicate weaker emotional reactions (n). High scores in psychoticism indicate loneliness, indifference, and difficulty adapting to the external environment (P), while low scores are normal (p).

Psychological flexibility was measured using the Acceptance and Action Questionnaire-II (AAQ-II), which contains 10 items. The AAQ-II adopts a Likert-style 7-point scoring system, with one point for complete non-compliance and seven points for complete compliance. Items 1, 6, and 10 were scored positively, and items 2, 3, 4, 5, 7, 8, and 9 were scored backwards. The sum of item scores is the total score of the scale, which ranges from 10 to 70 points. Higher scores indicate stronger psychological flexibility (A) and lower scores indicate weaker psychological flexibility (a). Cronbach's α coefficient for the scale was 0.705 (35).

Occupational stress was measured using a simplified version of the Occupational Stress Inventory-revised edition (OSI-R). We chose three dimensions of the OSI-R, including role overload (RO), psychological strain (PSY) and physical strain (PHS). A high RO score indicates a self-perceived high workload (R), whereas a low score indicates an acceptable workload (r). All the items were retained for each dimension to ensure the construct validity of the questionnaire. The simplified version of the OSI-R has good test-retest reliability and good homogeneity with the OSI-R (36).

### Statistical Analysis

STATA 14.0 software was used for analysis. Statistical analysis methods included partial correlation analysis, variance analysis, and fs-QCA. The fs-QCA evaluates the relationship between an outcome and all possible Boolean combinations of the predictors. The degree of contribution of each combination of predictors in a given outcome was assessed using Boolean logistic tests for solution consistency and total coverage of the outcome and combinations of multiple binary predictive risk factors (26, 37).

The basic steps of fs-QCA are as follows: (1) Use the setgen command to fuzzify the data to a range between 0 and 1 without changing the distribution of the original data; (2) Generate a set of combinations of independent variables and test the consistency of all combinations with the outcome (solution consistency); (3) Use the reduce command to reduce the factor combination set of the consistency test to obtain the final factor combination set that is meaningful or explanatory for the outcome, and calculate the overall coverage of the factor combination set (total coverage) (37).

In this study, the fs-QCA included a total of five independent variables: extroversion (E/e), neuroticism (N/n), psychoticism (P/p), psychological flexibility (A/a) and role overload (R/r). All independent variables were automatically divided into a high-score and a low-score group during the analysis process, where uppercase letters represent higher scores, and lowercase letters represent lower scores on the corresponding dimension.

## Results

### Occupational Stress of Medical Staff

Among the 1,801 participants, 357 were male (19.82%), and 1,444 (80.18%) were female. The age range of the subjects was 18–61 years old, with an average age of 30.9 ± 8.35 years. The PSY score of the participants was 26.8 ± 7.13, and the PHS score was 24.3 ± 6.50. The level of individual stress responses of the medical staff is shown in Table 1.

**Table 1:** The level of individual stress response of medical staff.

**Baseline variable**	***N* (%)**	**PSY**	**PHS**
**Sex**			
Male	357 (19.82)	26.2 ± 7.4	24.8 ± 7.0
Female	1,444 (80.18)	27.0 ± 7.1	24.1 ± 6.4
*P*		0.07	0.09
**Age**			
<25	456 (25.32)	26.2 ± 7.0	24.1 ± 6.2
25~	824 (45.75)	27.6 ± 7.2	24.8 ± 6.6
35~	375 (20.82)	26.8 ± 7.1	24.3 ± 6.7
45~	146 (8.11)	24.7 ± 6.5	21.8 ± 5.9
*P*		**<0.01**	**<0.01**
**Work experience**			
<10	1,204(66.9)	27.0 ± 7.1	24.5 ± 6.4
10~	328(18.2)	27.6 ± 7.7	24.7 ± 7.2
20~	269(14.9)	25.2 ± 6.2	22.5 ± 5.8
*P*		**<0.01**	**<0.01**
**Marital status**			
Single	770 (42.75)	26.6 ± 7.1	24.5 ± 6.4
Married	1,031 (57.25)	27.0 ± 7.1	24.1 ± 6.6
*P*		0.33	0.20
**Education**			
Below junior college	194 (10.77)	26.5 ± 6.7	24.1 ± 6.3
Junior college	829 (46.03)	26.7 ± 6.8	24.1 ± 6.2
Post-junior college	778 (43.20)	27.0 ± 7.5	24.5 ± 6.8
*P*		0.49	0.41

*PSY, psychological strain; PHS, physical strain. The bold values indicate a statistical difference in ANOVA*.

### Relationship Between Role Overload, Personality, Psychological Flexibility, and Stress

The partial correlation analysis among the included factors after adjusting for basic demographic characteristics is shown in Table 2. The results showed that there were statistically significant correlations between personality, psychological flexibility, role overload, and occupational stress (PSY and PHS) of the medical staff (*P* < 0.05). The correlation coefficients between neuroticism and PSY (0.552) and between psychological flexibility and PSY (0.520) were >0.50.

**Table 2:** Relationship between role overload, personality, psychological flexibility, and stress.

**Variable**	**PSY**	**PHS**
Extraversion	−0.336^a^	−0.256^a^
Neuroticism	0.552^a^	0.476^a^
Psychoticism	0.102^a^	0.151^a^
Psychological flexibility	−0.520^a^	−0.467^a^
Role overload	0.374^a^	0.410^a^

*Adjusted for age, sex, education, and marital status. PSY, psychological strain; PHS, physical strain*.

a *indicates P < 0.05*.

### High-Risk Factor Sets of Occupational Stress

To explore the high-risk factor sets of PSY and PHS among medical staff, we conducted an fs-QCA that included personality, psychological flexibility, and role overload. The setgen command was used to fuzzify the d/ata to a range between zero and one without changing the distribution of the original data. In addition, the independent variables were A/a (psychological flexibility), P/p (psychoticism), E/e (extroversion), N/n (neuroticism), and R/r (role overload). The fs-QCA was carried out with PSY and PHS as the dependent variables.

**Figure 1:**
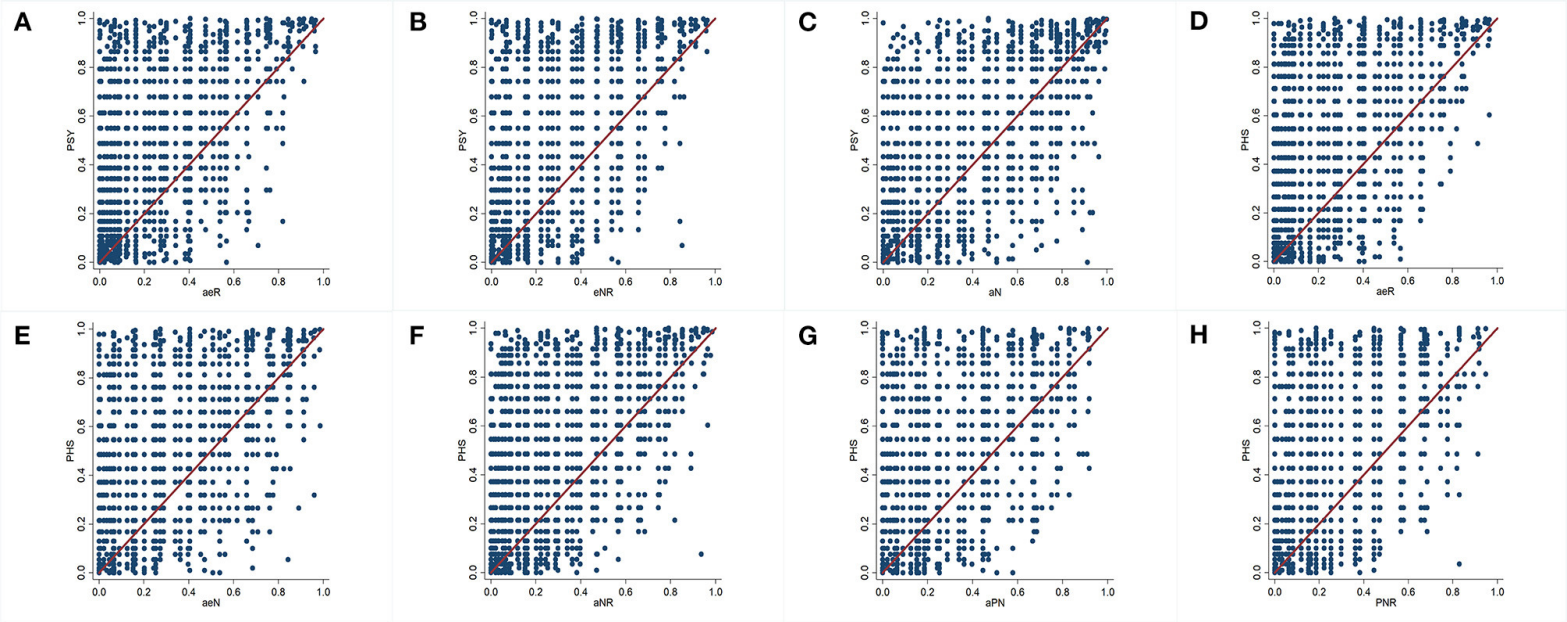
Sufficiency and necessity graph between different factor sets for psychological strain (PSY) and physical strain (PHS). The horizontal axis represents factor sets, and the vertical axis represents PSY or PHS. **(A)** Set “a*e*R” is a sufficient but not necessary condition for PSY. In other words, set “a*e*R” is a fuzzy subset of PSY. **(B)** Set “e*N*R” is a sufficient but not necessary condition for PSY and a fuzzy subset of PSY. **(C)** Set “a*N” is a sufficient but not necessary condition for PSY and a fuzzy subset of PSY. **(D)** Set “a*e*R” is a sufficient but not necessary condition for PHS and a fuzzy subset of PHS. **(E)** Set “a*e*N” is a sufficient but not necessary condition for PHS and a fuzzy subset of PHS. **(F)** Set “a*N*R” is a sufficient but not necessary condition for PHS and a fuzzy subset of PHS. **(G)** Set “a*P*N” is a sufficient but not necessary condition for PHS and a fuzzy subset of PHS. **(H)** Set “P*N*R” is a sufficient but not necessary condition for PHS and a fuzzy subset of PHS. E/e, extroversion/introversion; N/n, stronger emotional reactions such as anxiety, worry, and even irrational behavior/weaker emotional reactions; P/p, loneliness, indifference, and difficulty adapting to the external environment/normal; R/r, self-perceived high work pressure/self-perceived acceptable work pressure; A/a, stronger psychological flexibility/weaker psychological flexibility.

The consistency test showed that there were 12 common sets (apenR, apeNr, apeNR, apENr, apENR, aPenR, aPeNr, aPeNR, aPENr, aPENR, ApeNR and APeNR) with statistical significance in PSY (Table 3). The reduce command was used for the dimensionality reduction (Table 4). The results showed that low psychological flexibility score-introversion-high role overload (a^*^e^*^R), introversion-neuroticism-high role overload (e^*^N^*^R), and low psychological flexibility score-neuroticism (a^*^N) were high-risk factor sets for PSY. The above factor sets were sufficient, but not necessary for PSY (Figure 1). The coverage of different sets and the corresponding solution consistencies are listed in Table 4. The overall coverage and solution consistency values were 0.762 and 0.842, respectively.

**Table 3:** Consistency test for common sets of PSY and PHS.

**Set**	**YCons**	**N-consistency**			**Set value**		**Num bestfit**
		**NCons**	***F***	***P***	***F***	***P***	
**PSY**							
apenR	0.917	0.867	16.9	<0.001	1,085.5	<0.001	46
apeNr	0.923	0.804	71.2	<0.001	1,335.4	<0.001	85
apeNR	0.939	0.732	191.3	<0.001	1,762.0	<0.001	132
apENr	0.906	0.875	6.0	0.014	772.5	<0.001	40
apENR	0.932	0.817	76.2	<0.001	1,639.9	<0.001	49
aPenR	0.921	0.864	20.3	<0.001	1,041.3	<0.001	29
aPeNr	0.930	0.801	80.4	<0.001	1,304.7	<0.001	62
aPeNR	0.937	0.700	214.8	<0.001	1,704.5	<0.001	142
aPENr	0.903	0.868	6.8	0.009	684.8	<0.001	41
aPENR	0.925	0.788	94.8	<0.001	1,398.2	<0.001	76
ApeNR	0.916	0.865	16.8	<0.001	1,024.8	<0.001	31
APeNR	0.929	0.861	30.7	<0.001	1,262.9	<0.001	21
**PHS**							
apenR	0.903	0.868	7.2	0.007	881.7	<0.001	46
apeNr	0.896	0.840	15.6	<0.001	686.7	<0.001	85
apeNR	0.921	0.757	114.8	<0.001	1,300.2	<0.001	132
apENR	0.912	0.829	37.8	<0.001	1,137.3	<0.001	49
aPenR	0.905	0.855	13.7	<0.001	783.0	<0.001	29
aPeNr	0.908	0.829	31.4	<0.001	868.7	<0.001	62
aPeNR	0.922	0.711	167.6	<0.001	1,289.6	<0.001	142
aPENr	0.895	0.864	5.3	0.02	639.9	<0.001	41
aPENR	0.920	0.769	98.7	<0.001	1,204.7	<0.001	76
APeNR	0.916	0.868	14.3	<0.001	959.9	<0.001	21
APENR	0.901	0.871	5.3	0.02	710.1	<0.001	23

*PSY, psychological strain; PHS, physical strain; E/e, extroversion/introversion; N/n, stronger emotional reactions such as anxiety, worry, and even irrational behavior/weaker emotional reactions; P/p, loneliness, indifference, and difficulty adapting to the external environment/normal; R/r, self-perceived high work pressure/self-perceived acceptable work pressure; A/a, stronger psychological flexibility/weaker psychological flexibility. The set value is 0.70*.

**Table 4:** Dimensionality reduction for common sets of PSY and PHS.

**Set**	**Raw coverage**	**Unique coverage**	**Solution consistency**	**Num bestfit**
**PSY**				
a*e*R	0.509	0.047	0.905	349
e*N*R	0.506	0.044	0.905	326
a*N	0.671	0.209	0.861	627
**PHS**				
a*e*R	0.500	0.046	0.888	349
a*e*N	0.531	0.032	0.876	421
a*N*R	0.547	0.027	0.893	399
a*P*N	0.491	0.017	0.878	321
P*N*R	0.471	0.043	0.890	262

*PSY, psychological strain; PHS, physical strain; E/e, extroversion/introversion; N/n, stronger emotional reactions such as anxiety, worry, and even irrational behavior/weaker emotional reactions; P/p, loneliness, indifference, and difficulty adapting to the external environment/normal; R/r, self-perceived high work pressure/self-perceived acceptable work pressure; A/a, stronger psychological flexibility/weaker psychological flexibility. The total coverage and solution consistency of PSY are 0.762 and 0.842, respectively. The total coverage and solution consistency of PHS are 0.731 and 0.835, respectively*.

The consistency test showed that there were 11 common sets (apenR, apeNr, apeNR, apENR, aPenR, aPeNr, aPeNR, aPENr, aPENR, ApeNR, and APENR) with statistical significance in the PHS (Table 3). The reduce command was used for the dimensionality reduction (Table 4). The results showed that low psychological flexibility score-introversion-high role overload (a^*^e^*^R), low psychological flexibility score-introversion-neuroticism (a^*^e^*^N), low psychological flexibility score-neuroticism-high role overload (a^*^N^*^R), low psychological flexibility score-psychoticism-neuroticism (a^*^P^*^N), and psychoticism-neuroticism-high role overload (P^*^N^*^R) were high-risk factor sets for PHS. The above factor sets were sufficient, but not necessary for PHS (Figure 1). The coverage of different sets and the corresponding solution consistencies are listed in Table 4. The overall coverage and solution consistency values were 0.731 and 0.835, respectively.

## Discussion

Medical staff are a high-risk group for occupational stress and a key population that should be considered in stress interventions. A systematic review focused on the mental health of medical staff showed that the overall prevalence of occupational stress ranged from 29.8 to 63.0%, with more severe symptoms among nurses, and female and young workers (13). During the COVID-19 pandemic, medical staff faced greater pressure and challenges than ever. At the beginning of the COVID-19 pandemic, reports stated that the scores of medical staff on various indicators of stress were significantly higher than those of the control group (38, 39). One year after the COVID-19 pandemic, a survey of medical staff in Saudi Arabia reported widespread symptoms of depression, anxiety, and stress (40).

It is generally believed that demographic factors, social psychological factors, and personality are related to occupational stress (15, 19), while excessive workload, task conflicts, and exposure to occupational hazards in the workplace are risk factors for stress (18, 41, 42). In this study, we found that personality, psychological flexibility, and role overload were associated with PSY and PHS among the medical staff. Neuroticism, psychoticism, and RO were positively correlated with occupational stress, whereas psychological flexibility and extraversion were negatively correlated with occupational stress. The results of the partial correlation analysis were consistent with those of previous studies that focused on the individual effects of specific factors (9).

From a sociological perspective, individuals are complex combinations of various characteristics. If we want to conduct interventions on occupational stress, we should first determine the combination of traits that are more prone to stress. This study further conducted in-depth discussions on occupational stress with the help of the fs-QCA. The results showed that low psychological flexibility score-introversion-high role overload, introversion-neuroticism-high role overload, and low psychological flexibility score-neuroticism are high-risk for PSY, while low psychological flexibility score-introversion-high role overload, low psychological flexibility score-introversion-neuroticism, low psychological flexibility score-neuroticism-high role overload, low psychological flexibility score-psychoticism-neuroticism, and psychoticism-neuroticism-high role overload are high-risk for PHS. The above factor sets were sufficient but not necessary conditions for occupational stress. This means that individuals with a combination of the above factors are more likely to suffer from stress than those in the non-carrier group.

During the COVID-19 pandemic, medical staff are at a higher risk than ever for mental health issues, such as occupational stress, anxiety, and burnout. For occupational stress interventions and psychological counseling, targeted and individualized measures should be taken according to the specific characteristic combinations of different individuals. In fact, a unified and universal intervention method is insufficient to fully deal with the mental health problems currently faced by the medical staff. In addition, in terms of job suitability and human resource management, the conclusions based on fs-QCA have certain benefits.

Despite its important findings, this study has some limitations. First, the cross-sectional nature of this article that prevents inference on a casualty. From an epidemiological point of view, the observed associations in our study cannot be interpreted in a causal sense. Second, the study only included role overload, personality characteristics, and psychological flexibility for fs-QCA, and did not include other factors and dimensions of occupational stress. It should be noted that including too many independent variables complicates the interpretation of the fs-QCA results, which is one of the inherent limitations of QCA. Further, fs-QCA, as a qualitative analysis method, cannot provide the quantitative effect of a single independent variable.

## Conclusion

Based on the fs-QCA strategy, this study conducted an innovative approach and attempted to explore the high-risk factor set of occupational stress in medical staff, instead of being limited to the study of single-factor effects. This study provides new strategies and ideas for the exploration of occupational stress risk factors and formulation of intervention measures for medical staff during the COVID-19 pandemic.

## Data Availability Statement

The raw data supporting the conclusions of this article will be made available by the authors, without undue reservation.

## Ethics Statement

The studies involving human participants were reviewed and approved by Ethics Committee of the West China School of Public Health, Sichuan University (No. Gwll2021070). The patients/participants provided their written informed consent to participate in this study.

## Author Contributions

LH, YLa, and LQ designed the study. LQ and YZ analyzed the data and drafted the manuscript. LQ, YZ, FJ, YLi, YLa, and LH edited the manuscript. All authors contributed to the article and approved the submitted version.

## Funding

This study was supported by the National Nature Science Foundation of China (No. 82073521), Scientific research fund for young teachers of Sichuan University (No. 2015SCU11009), Scientific research project of Health Commission of Sichuan Province (No. 17PJ451), and Scientific research project of Department of Education of Sichuan Province (No. 17ZB0247).

## Conflict of Interest

The authors declare that the research was conducted in the absence of any commercial or financial relationships that could be construed as a potential conflict of interest.

## Publisher's Note

All claims expressed in this article are solely those of the authors and do not necessarily represent those of their affiliated organizations, or those of the publisher, the editors and the reviewers. Any product that may be evaluated in this article, or claim that may be made by its manufacturer, is not guaranteed or endorsed by the publisher.
